# Patient safety and climate change: findings from a cross-sectional survey in Germany

**DOI:** 10.1186/s12889-024-20752-x

**Published:** 2024-11-21

**Authors:** Olga Amberger, Dorothea Lemke, Anette Christ, Hardy Müller, David Schwappach, Max Geraedts, Beate S. Müller

**Affiliations:** 1 Institute of General Practice, Goethe University Frankfurt, Theodor-Stern-Kai 7, 60590 Frankfurt am Main, Germany; 2 Gesundheitsamt Frankfurt Am Main, Breite Gasse 28, 60313 Frankfurt Am Main, Germany; 3 German Society for Patient Safety, August-Bebel-Straße 13, 72762 Reutlingen, Germany; 4 Institute of Social and Preventive Medicine (ISPM), University Bern, Mittelstrasse 43, 3012 Bern, Switzerland; 5 Institute for Health Services Research and Clinical Epidemiology, University of Marburg, Karl-Von-Frisch-Straße 4, 35043 Marburg, Germany; 6 Institute of General Practice, University of Cologne, 50937 Cologne, Germany

**Keywords:** Climate change, Patient safety, Public Health

## Abstract

**Background:**

Patient safety has become a priority issue in health policy strategies in Germany in the last several years, and is especially important in the era of climate change. This study aimed to assess public perceptions about the patient safety impact of climate change and the demographic and socioeconomic factors influencing patient perception in Germany.

**Methods:**

A cross-sectional study was conducted in Germany in 2023, using data from the TK Monitor of Patient Safety. The TK Monitor of Patient Safety is a national survey of the population on the state of safety in medical care. Self-reported data were collected from 1,000 randomly selected adults living in Germany. Demographic and socioeconomic variables were regressed on climate change perception using an ordinal logistic regression approach.

**Results:**

Our results revealed that half of respondents are concerned about climate change affecting their health and 40% of the respondents would like to have climate-sensitive health counseling by their general practitioner. The results showed that demographic variables, such as gender and age, and socioeconomic variables, such as education level and income, are important factors influencing the perception of climate change-related patient safety risks. However, no association was found between urban/rural residence and patient perception.

**Conclusions:**

Our study highlights patient safety as a public health concern in the era of climate change. The German public appears to view climate change as harmful to patient safety. Our findings also show that it is necessary to carry out diagnoses focused on demographic and socioeconomic factors to determine which aspects should be strengthened through programs aimed at reducing patient safety risks associated with climate change.

**Supplementary Information:**

The online version contains supplementary material available at 10.1186/s12889-024-20752-x.

## Introduction

Patient safety continues to have a position high on the international agenda [1] in times of multiple crises, especially in the face of climate change [2]. Globally, an estimated one in ten patients is harmed in health care and more than three million deaths occur annually due to unsafe care [3]. Patient harm reduces global economic growth by 0.7% a year. The patient safety “epidemic” is coinciding with global climate change, which has a profound impact on nearly every aspect of health. It is negatively impacting patients' physical and mental health [4]. In Germany in 2022, more than 8,000 people died from the consequences of severe hot weather [5]. Extreme heat waves can cause acute myocardial and cerebrovascular events, and fatigue, and can aggravate chronic diseases such as kidney diseases, and type II diabetes. Respiratory morbidity and mortality are on the rise due to increases in air pollution [6], severe wildfires, dust storms, extended heat waves, and floods [7]. Moreover, climate change is affecting transmission and spread of vector-borne diseases such as dengue, Zika and West Nile fever, which first appeared in Germany in 2019 [8].

Several studies have examined individuals’ perceptions of risk, and knowledge of climate change [9–11]. Pidgeon [10] noted that awareness of climate change is increasing in society. Hathaway et al. [9] stressed that whilst most residents of developed countries know little or nothing about the health relevance of climate change, among vulnerable publics in developing nations, the perception that climate change is harming health appears to be high. In spite of recognition of the health harms of climate change, there is still little known about the patient safety risks associated with climate change [12]. In a narrative review [13], Al-Marwani highlights a range of safety gaps including increasing patient vulnerability, shortage of drugs, increasing occurrence of medical errors, and delay in cancer detection and management. Climate change revealed the loss of patients' access to healthcare facilities, impaired service delivery due to disruption of supply chains resulting from extreme climate events as well as employee shortages [14]. While it seems evident that climate change will add to patient safety hazards, little is known about public perceptions related to the patient safety impacts of climate change.

Studies examining climate change perception and connections to demographic and sociocultural variables are highly valuable for developing adaptive measures to climate change and policies for its sustainable development [15]. Despite the importance of this research, there is a lack of focus on identifying socioeconomic and demographic factors that affect the public perception of patient safety risks associated with climate change. Identifying these factors is crucial for designing targeted strategies to tackle patient safety problems in the face of climate change. For instance, if age is found to significantly influence perceptions, initiatives in education in climate change and patient safety, tailored to different age groups, could be implemented.

This study, therefore, aimed to (i) provide data on the public perception of patient safety risks associated with climate change, and (ii) examined the demographic and socioeconomic factors influencing this perception.

## Methods

### Data

The primary data source is the Techniker Krankenkasse (TK) Monitor of Patient Safety, a national annual survey that was established to help understand trends in patient safety from the German public’s point of view. The study design has been described in full elsewhere [16]. Briefly, the TK Monitor of Patient Safety is a large national survey. It is undertaken yearly and collects population-related data on perceptions, experience, and knowledge pertaining to patient safety from 1,000 randomly selected inhabitants via computer assisted telephone interviews (CATI). The random sample is herein taken from the population of the residential population. Every year, the survey is updated to include questions about special issues. The first survey took place from October to November 2019. The follow-up surveys were conducted in August 2020, in June 2021, in April to May 2022, and in June 2023. Selected results from the surveys have been published elsewhere [17–19]. The data reported here are from 2023.

The checklist for Strengthening the Reporting of Observational Studies in Epidemiology (STROBE) was used as a reporting guideline (see online supplementary file 1).

### Participants

Self-reported data from a random sample of 1,000 adults from the population of the residential population were collected. Inclusion criterion was 18 or more years of age (70.37 million adults living in Germany in 2023). Exclusion criterion was non-German speakers (5% households with not sufficient German language skills). The representativeness of the sample was ensured by drawings of ADM (Arbeitskreis Deutscher Marktforscher, Association of German Market and Social Research Institutes) samples [20] and by comparison with the data of the German Federal Statistical Office [21]. The ADM sampling system is based on the dual-frame method, in which a random sample of landline and mobile telephone numbers are used. This sampling method provides almost complete coverage of the German population [22]. We herein employed a multi-level stratified random sampling procedure with 70% landline, and 30% mobile phone numbers. The proportion of households with a landline telephone connection is around 83% in Germany and 82.2% of the population are smartphone users. For respondent selection within a household we used the last-birthday method. In this method, the selected respondent is the person in the household with the birthday that has most recently occurred. The survey was anonymous. Participation in the study was voluntary and no expense allowance was paid. All participants gave their informed consent. Data were collected by the Society for Social Research and Statistical Analysis Ltd. (forsa) [23] according to the German law of data protection. The contact data and quotas including response rate were deleted immediately after the interviews.

Ethical approval was not sought for this study as general public survey studies are not required to undergo ethical review in Germany [24]. The forsa institute that conducted the survey signed the international ethics code for public opinion research (ICC/ESOMAR Code). The TK Monitor of Patient Safety was financed by the statutory health fund ‘Techniker Krankenkasse’ (award/grant number: Not applicable).

### Questionnaire

The TK Monitor of Patient Safety questionnaire was developed in collaboration with patient safety researchers, social scientists, medical practitioners, health scientists, and psychologists. Questions and response items were based on existing questionnaires described in the literature [25–28], and discussions among the questionnaire developers. Some of the response items on factors associated with patient safety were obtained from preliminary work [27, 28]. The questionnaire was pre-tested and validated by a panel of experts in survey methodology. Some minor adjustments to the questions were made.

The questionnaire consisted of closed questions divided into three sections (see online supplementary file 2) preceded by a brief introduction explaining why the survey was being undertaken. In section (A) questions on perceptions, experiences, and subjective information relating to patient safety in medical care were scored on a Likert scale, from very likely to unlikely. The questions in section (B) covered perceptions and knowledge regarding climate change. In section (C) sociodemographic and socioeconomic data were collected. Section A and C remained almost unchanged every year, while section B changed from survey to survey. The data reported here are from sections B and C.

### Data analysis

Descriptive statistics were obtained using the R software [29]. Data were weighted for gender, age, education level, and area (metropolitan/rural) population distribution. Weighting was based on iterative proportional fitting. Ordinal logistic regression was used to determine the factors influencing respondents’ perception about climate change, and odds ratios (OR) and 95% confidence intervals (CI) were calculated. Model covariates included a combination of demographic and socioeconomic characteristics obtained from survey responses. We included the following self-reported characteristics: age (18–39, 40–59, and ≥ 60 years); gender; education level (up to secondary school, tertiary/university, and not specified), area (rural area with < 20,000 residents, suburban area ranging from 20,000–99,999 residents, urban area with ≥ 100,000 residents), perceived well-being (very good/good, fair/poor/very poor, and not specified), and monthly income amount (< 3,000, ≥ 3,000 euros, and not specified). The monthly income amount was used as a measure of economic status. We originally intended to include self-reported professional positions, and marital status/household structure as covariates, but 50% of survey respondents did not answer these questions. All tests were performed two-sided at the 0.05 significance level.

## Results

### Participants

The main sociodemographic characteristics of participants are presented in Table 1. The sample was representative for the German population regarding age, gender, educational level, and insurance status compared with the data of the German Federal Statistical Office [21].

**Table 1:** Sociodemographic characteristics of participants and the German population in 2023 [21]

Demographic	Characteristics	Participants N = 1000	German population
**Gender**	Male	489 (48.9%)	49.3%
	Female	511 (51.1%)	50.7%
**Age**	18–39 years	315 (31.5%)	28.0%
	40–59 years	332 (33.2%)	27.2%
	≥ 60 years	352 (35.2%)	30.8%
**Employment status**	Employed	516 (51.6%)	77.0%
	Unemployed (economically inactive)	484 (48.4%)	23.0%
**Chronic condition**	Yes	339 (33.9%)	40.0%
	No	660 (66.0%)	60.0%
**Perceived well-being**	Very good/good	578 (57.8%)	65.0%
	Fair/poor/very poor	422 (42.2%)	35.0%
**Prescription medication**	No	473 (47.3%)	50.0%
	Yes	527 (52.7%)	50.0%
**Educational level**	Up to secondary school	565 (56.5%)	66.0%
	Tertiary/university	382 (38.2%)	30.0%
**Area**	Rural area (< 20,000 residents)	380 (38.0%)	8.0%
	Suburban area (20,000–99,999 residents)	277 (27.7%)	17.0%
	Urban area (≥ 100,000 residents)	343 (34.3%)	78.0%
**Insurance status**	Statutory health insurance	875 (87.5%)	87.0%
	Private health insurance	125 (12.5%)	13.0%

### Perception about climate change impact on patient safety

Half of respondents (51%) were very or extremely concerned about climate change affecting their health (Table 2); only 13% were not concerned. Of the respondents, 40% would like to be advised by their general practitioner (GP) whether there is a health risk for them personally due to climate change and how they can better protect their health in heat waves (climate-sensitive health counseling). Eight percent of respondents thought on several occasions, and 5% once, that they needed medical treatment due to hot weather. The majority of respondents estimated that heat waves could have a negative impact on staff performance (92% agreement), chronic illnesses (87%), mental health (71%), medication (66%) and wound healing (65%). Half of the respondents (53%) considered the hospitals in Germany to be well or very well prepared to guarantee safe care even during a heat wave (item 5). In contrast, 45% were of the opinion that German hospitals are less well or poorly prepared during heat waves.

**Table 2:** Participants’ perceptions about climate change impact on patient safety

Item	Overall	Gender		Age			Perceived well-being		Educational level		Economic status	
		**Male**	**Female**	**18–39 years**	**40–59 years**	** ≥ 60 years**	**Very good/good**	**Fair/poor/very poor**	**Up to secondary school**	**Tertiary/university**	** < €3,000**	** ≥ €3,000**
**Concerns about climate change**												
Very serious/serious/some concerns	86%	86%	87%	89%	87%	83%	84%	89%	84%	89%	83%	82%
No concerns/Not specified	13%	15%	13%	11%	13%	17%	16%	11%	16%	11%	17%	12%
**Wish to have climate-sensitive health counseling by the general practitioner**												
Yes, definitely/yes, probably	40%	36%	44%	39%	34%	44%	33%	48%	44%	33%	48%	35%
Probably not/certainly not	61%	64%	57%	61%	65%	55%	67%	51%	56%	67%	52%	66%
**Need for medical treatment due to hot weather**												
Yes, often/yes, once	13%	8%	16%	22%	10%	8%	6%	22%	11%	10%	12%	11%
No	87%	91%	84%	79%	90%	92%	94%	78%	89%	89%	88%	88%
**Negative impact of heat waves on…**												
Staff performance in ambulatory care	92%	91%	92%	92%	92%	88%	92%	91%	89%	95%	88%	94%
Hospital staff performance	92%	89%	94%	90%	94%	90%	92%	91%	89%	94%	88%	94%
Chronic illnesses	87%	85%	89%	85%	89%	86%	86%	88%	87%	88%	88%	86%
Mental health	71%	76%	67%	72%	67%	72%	68%	75%	69%	76%	75%	68%
Medication	66%	63%	69%	63%	69%	62%	68%	64%	64%	70%	65%	69%
Wound healing	65%	63%	67%	63%	67%	53%	66%	64%	60%	71%	61%	69%
**Preparation of hospitals during heat wave**												
Very good/good	53%	57%	49%	69%	67%	55%	52%	54%	57%	48%	57%	50%
Less well/poorly/not specified	46%	44%	51%	30%	33%	45%	48%	46%	43%	51%	43%	50%

### Demographic and socioeconomic determinants of perception of climate change-related patient safety risks

Tables 3–4 show the results of the ordinal logistic regression models. Demographic and socioeconomic variables influenced the perception of climate change-related patient safety risks. Female respondents, compared to male respondents, had a higher chance of having concerns about climate change. There was also a higher chance among female respondents of perceiving heat-related health risk, and poor wound healing during heat waves as well as the perception regarding robust heat preparedness of hospitals. Middle-age adults (40–59 years), compared to young adults (18–39 years of age), had a higher chance to perceive poor staff performance in ambulatory care during heat waves and a lower chance to perceive heat-related health risk. Respondents aged 60 years and older, compared to young adults, had a lower chance to perceive both heat-related health risk and poor wound healing during heat waves. Respondents with higher education, compared to those with education up to secondary school, had a higher chance to perceive poor staff performance in ambulatory care, as well as poor wound healing, and mental health during heat waves. Respondents with unspecified education, compared to those with education up to secondary school had a higher chance to perceive heat-related health risk. Respondents with high income, compared to those with low income, had a lower chance to desire climate-sensitive health counseling from a general practitioner. Respondents with unspecified income, compared to those with low income, had a lower chance to desire climate-sensitive health counseling and to perceive a negative impact of heat waves on medication. Finally, respondents with poor perceived well-being, compared to those with a very good or good well-being, had a higher chance to desire climate-sensitive health counseling from a general practitioner, to perceive heat-related health risk, and robust preparedness of hospitals. Urban or rural residence was not associated with perceptions of climate change-related patient safety risks.

**Table 3:** Demographic and socioeconomic determinants of participants’ perception of climate change-related patient safety risks

		**Climate change health concerns**^**1**^			**Climate-sensitive health counseling by GP**^**2**^				**Heat-related health risk**^**3**^			**Robust hospital preparedness for heatwaves**^**4**^		
*Predictor*	*Reference*	*OR*	*CI*	*p-value*	*OR*	*CI*	*p-value*		*OR*	*CI*	*p-value*	*OR*	*CI*	*p-value*
**Age**														
< 60 years	18–39 years	0.88	0.64 – 1.21	0.443	0.89	0.63 – 1.25		0.487	0.55	0.32 – 0.94	**0.028**	1.09	0.79 – 1.50	0.597
≥ 60 years		1.06	0.76 – 1.47	0.726	1.25	0.89 – 1.76		0.201	0.31	0.17 – 0.54	** < 0.001**	1.08	0.77 – 1.51	0.652
**Gender**														
Female	Male	1.37	1.06 – 1.78	**0.016**	1.02	0.78 – 1.34		0.868	2.12	1.34 – 3.39	**0.001**	1.33	1.02 – 1.73	**0.034**
**Well-being**														
Poor	Very good/ good	1.25	0.95 – 1.63	0.112	1.44	1.09 – 1.90		**0.010**	4.64		** < 0.001**	1.36	1.04 – 1.79	**0.027**
**Educational level**														
Tertiary/ university	Up to secondary school	1.32	0.99 – 1.76	0.059	0.93	0.69 – 1.25		0.609	1.08	0.67 – 1.78	0.758	1.31	0.98 – 1.75	0.072
Not specified		0.73	0.26 – 1.93	0.528	1.19	0.42 – 3.16		0.736	4.10	1.10 – 13.43	**0.025**	0.77	0.27 – 2.04	0.610
**Economic status**														
≥ €3,000	< €3,000	0.95	0.71 – 1.27	0.726	0.61	0.45 – 0.81		**0.001**	0.70	0.43 – 1.13	0.141	1.16	0.87 – 1.56	0.307
Not specified		1.08	0.68 – 1.73	0.732	0.53	0.32 – 0.86		**0.011**	1.24	0.58 – 2.49	0.561	1.00	0.61 – 1.61	0.991
Area*														
Suburban	Rural	0.86	0.63 – 1.18	0.349	0.84	0.61 – 1.17		0.309	1.02	0.60 – 1.72	0.935	1.30	0.95 – 1.78	0.107
Urban		1.31	0.97 – 1.77	0.083	0.78	0.56 – 1.06		0.116	0.71	0.41 – 1.22	0.220	1.08	0.79 – 1.46	0.632

*CI* confidence interval, *GP* general practitioner, *OR* odds ratios.

^1^Are you concerned about climate change having an impact on your health?

^2^Would you like your family doctor to advise you whether there is a health risk for you personally due to climate change and how you can better protect your health during heat waves?

^3^Have you experienced that your health was so bad because of the hot weather that you thought you needed medical treatment?

^4^Are hospitals in Germany well prepared to ensure safe care even during a heat wave?

*Rural area < 20,000 residents, suburban area ranging from 20,000–99,999 residents, urban area ≥ 100,000 residents.

**Table 4:** Demographic and socioeconomic determinants of participants’ perception of negative impact of heat waves on different health areas^1^

		**Medication**			**Chronic illnesses**			**Hospital staff performance**			**Staff performance in ambulatory care**			**Wound healing**			**Mental health**		
*Predictor*	*Reference*	*OR*	*CI*	*p-value*	*OR*	*CI*	*p-value*	*OR*	*CI*	*p-value*	*OR*	*CI*	*p-value*	*OR*	*CI*	*p-value*	*OR*	*CI*	*p-value*
**Age**																			
< 60 years	18–39 years	0.74	0.52 – 1.05	0.096	1.44	0.90 – 2.31	0.126	1.75	0.93 – 3.36	0.087	2.07	1.04 – 4.33	**0.044**	1.02	0.72 – 1.44	0.916	0.98	0.68 – 1.39	0.890
≥ 60 years		0.71	0.50 – 1.02	0.064	1.21	0.76 – 1.95	0.420	0.92	0.52 – 1.63	0.783	0.75	0.42 – 1.34	0.342	0.63	0.45 – 0.89	**0.009**	1.08	0.75 – 1.57	0.677
**Gender**																			
Female	Male	1.10	0.83 – 1.46	0.502	1.30	0.89 – 1.92	0.181	1.62	1.00 – 2.68	0.053	1.40	0.85 – 2.32	0.189	1.42	1.08 – 1.88	**0.012**	0.82	0.61 – 1.10	0.180
**Well-being**																			
Poor	Very good/ good	1.00	0.75 – 1.34	0.998	1.19	0.80 – 1.79	0.406	0.87	0.53 – 1.43	0.578	0.99	0.60 – 1.66	0.977	0.96	0.72 – 1.28	0.785	1.20	0.88 – 1.63	0.246
**Educational level**																			
Tertiary/ university	Up to secondary school	1.13	0.83 – 1.54	0.427	1.35	0.89 – 2.04	0.156	1.53	0.91 – 2.53	0.103	1.84	1.09 – 3.10	**0.021**	1.44	1.06 – 1.93	**0.018**	1.68	1.22 – 2.29	**0.001**
N.s		0.84	0.31 – 2.38	0.727	0.68	0.23 – 2.52	0.523	1.82	0.35 – 33.56	0.570	0.90	0.23 – 5.98	0.899	1.06	0.40 – 3.01	0.906	0.87	0.33 – 2.46	0.786
**Economic status**																			
≥ €3,000	< €3,000	1.05	0.77 – 1.43	0.758	0.90	0.58 – 1.37	0.618	1.14	0.68 – 1.90	0.612	1.29	0.76 – 2.16	0.340	1.12	0.82 – 1.51	0.475	0.76	0.55 – 1.06	0.107
N.s		0.47	0.29 – 0.75	**0.002**	1.05	0.54 – 2.19	0.891	1.58	0.68 – 4.35	0.326	2.06	0.83 – 6.24	0.152	0.83	0.52 – 1.35	0.451	0.80	0.48 – 1.37	0.412
Area*																			
Suburban	Rural	0.90	0.64 – 1.26	0.538	0.80	0.51 – 1.25	0.321	1.11	0.61 – 2.03	0.741	0.96	0.54 – 1.73	0.895	1.09	0.78 – 1.52	0.625	1.04	0.74 – 1.48	0.803
Urban		0.96	0.69 – 1.33	0.805	1.06	0.67 – 1.68	0.806	0.96	0.55 – 1.67	0.872	1.26	0.70 – 2.32	0.444	1.20	0.87 – 1.66	0.256	1.26	0.90 – 1.78	0.178

*CI* Confidence interval, *N.s*. not specified, *OR* odds ratios.

^1^Can heat waves have a negative impact on the following areas?

*Rural area < 20,000 residents, suburban area ranging from 20,000–99,999 residents, urban area ≥ 100,000 residents.

## Discussion

This study showed a high perception of the health threat of climate change among the general German population. Our study is the first study assessing perceptions, experiences, and knowledge relating to patient safety and climate change in Germany.

The respondents expressed a high level of awareness of the climate change impact on patient safety. A majority was concerned about their own health with regard to climate change and suspect a negative impact of heat waves on various aspects of health care. In a literature review by Hathaway et al. [9], findings are mixed. While among vulnerable publics in Asia and Africa, awareness of increasing climate change-related health harms is high, most Americans and Canadians have little self-assessed knowledge and risk perception. However, when asked specific closed-ended questions about the topic [30], the perception that climate change is harming health appears to be much higher than people’s answers to open-ended questions. The authors of the review [9] gave two possible explanations for the discrepancy between findings. One is that the public has more knowledge than open-ended responses suggest, but because the issue is salient to many people, specific health impacts do not come to mind unless specifically asked. Another explanation may be that many people do not have prior perceptions of climate change as a health issue, but when asked closed-ended questions, they draw on general negative views on the topic. In our study, a considerable proportion of the respondents expressed their desire for specific advice from their GP. Indeed, as public health advocates, GPs have an important role in climate change adaptation [31]. However, there is a gap between the willingness of GPs to deal with health risks due to climate change and their lack of knowledge of effective interventions [32]. The German Association of General Practitioners (Hausärztinnen- und Hausärzteverband) has issued a manual for GPs to deal with the implications of climate change [33]. In our results, slightly less than half of the respondents expressed doubts about hospitals’ ability to adapt to heat wave periods. A recent survey of the German Hospital Institute suggests that these doubts are justified [34]. Only 56% of hospitals intend to plan heat regulation measures such as shading and active cooling, and only 17% of facilities actually have a heat action plan or an emergency plan for heat events. Thus, regardless of facility size, German hospitals are not well prepared for extreme weather events and especially for heat waves. Moreover, existing measures are currently not presented transparently [35].

This study found that demographic and socioeconomic variables influenced the perception of climate change-related patient safety risks, whereas no association was found between urban or rural residence and patient perception. This finding agrees with a survey from Lee et al. [11], who employed data collected on public perception of climate change from 119 countries. Moreover, it was found that the level of education played a crucial role in shaping perceptions of climate change risks in 62% of countries globally [11]. The level of education can impact public perception on climate change, as individuals with higher educational levels are more inclined to comprehend intricate climate change issues compared to those with lower levels of education. Reames et al. [15] have demonstrated that health concern was associated with individual- and area-level covariates, like age, sex, air quality alert knowledge, O_3_ exposure, and poverty. The evidence on the association between place of residence and climate attitudes appears to be mixed. In our study, urban or rural residence was not associated with perceptions of climate change-related patient safety risks. A study conducted in Pakistan found that people in rural areas were less concerned about environmental issues than those in urban areas [36]. However, the level of concern depended on the specific environmental problem; for example, farmers were more concerned about issues related to agriculture [37]. Among the European population, rural citizens were often more likely to deny anthropogenic climate change [38] and showed less support for climate policies [39]. Other studies have found that environmental attitudes are influenced by place identity, which refers to the connection between a person’s sense of self and their attachment to a specific place [40, 41]. Hence, future research should explore how identification with one’s district relates to the perception of climate change-related patient safety risks among urban and rural residents.

### Strengths and limitations of the study

The following aspects should be considered when interpreting the results. The questionnaire was based on revision of scientific literature in the field of patient safety but has not been validated in research studies. However, the selection of survey questions and response items underwent a thorough development process to ensure that its content, structure and wording would be appropriate for the respondents. The content was discussed with leading German patient safety researchers. Participants may have been influenced by social desirability bias that may result in more positive accounts of patient safety issues than are actually the case. However, the questions generally did not concern the participants’ attitudes concerning patient safety, but rather investigated their perceptions. Furthermore, as the contact database was deleted immediately after the information had been collected, in accordance with data protection regulations, the response rate and reasons for non-participation are unknown. This has implications for the generalizability of the findings. The views of people who do and do not participate may herein differ. However, the study population corresponds to that of the total population in the following aspects age, gender, educational level, and insurance status; the sample size was large and statistically robust. Moreover, in our study, the focus was on subjective views.

The strength of this study lies in its representative nature, and the large random sample. For the first time, data on perceptions, experience, and knowledge relating to patient safety impact of climate change were collected in a public sample. Moreover, the results reflect the views of the general public and provide important knowledge about current patient safety trends in Germany. The CATI interviewing technique prevents measurement errors [42] and item nonresponse [43].

## Conclusions

This is the first study assessing public perceptions of patient safety risks associated with climate change and their demographic and socioeconomic factors in Germany. The results illustrate, firstly, a high awareness of climate change impact on health among the German public. Secondly, demographic and socioeconomic variables are important factors influencing public perception about climate change and patient safety. Moreover, our findings indicate that the different age ranges, education levels and income of the population must be taken into account when addressing the perception of certain patient safety risks. Thus, identifying the degree of influence of demographic and socioeconomic variables on the perception of health-related climate change risk is a first step to designing specific action strategies to address these problems.

## Supplementary Information

Supplementary Material 1.

Supplementary Material 2.

## Data Availability

The datasets used and analysed during the current study are available from the corresponding author on reasonable request.

## Abbreviations

ADM: Arbeitskreis Deutscher Marktforscher, Association of German Market and Social Research Institutes
CATI: Computer assisted telephone interviews
CI: Confidence interval
GP: General practitioner
OR: Odds ratios
STROBE: Strengthening the Reporting of Observational Studies in Epidemiology
TK: Techniker Krankenkasse
