# Supplementary material for: Patient safety and climate change: findings from a cross-sectional survey in Germany
Source: BMC Public Health. 2024 Nov 21;24:3233. doi: 10.1186/s12889-024-20752-x (PMC11580531; doi:10.1186/s12889-024-20752-x)
Supplement: Supplementary file 1 — Supplementary Material 1. [file 12889_2024_20752_MOESM1_ESM.pdf]

Questionnaire TK Monitor of Patient Safety 2023

Section A
Standard introduction

Question S1
What is your year of birth?

Question S2
Gender of the interviewee.

1 male
2 female
3 non-binary
4

Question A
Health insurance
What is the form or name of your statutory health insurance provider? Or do you have private health insurance?

1 private health insurance (PKV, no matter what health insurer)
2 AOK (Allgemeine Ortskrankenkasse)
3 Barmer (also: Barmer GEK, BEK)
4 BKK – health insurance provider for employees at certain companies
5 DAK – (Deutsche Angestellten-Krankenkasse, also DAK Health)
6 IKK – health insurance provider for employees at certain companies
7 KKH (Kaufmännische Krankenkasse)
8 Knappschaft ("miner's health insurance provider")
9 TK - Techniker Krankenkasse
10 other statutory health insurance provider (i.e. NOT private insurer)
11 do not know/not specified ➔ END, no interview

Filter: only for persons with statutory health insurance (see question A items 2-10)
Question B
Health insurance status
And what is your health insurance status: Are you compulsorily insured, voluntarily insured or co-insured as a family member with your health insurance fund?

1 compulsory health insurance
2 voluntary insurance (has statutory health insurance despite fulfilling financial requirements to be eligible for private health insurance)
3 co-insured as a family member
4 do not know/not specified

To all

Question 1 (only in 2019)

When you think of the term 'patient safety', which of the following aspects do you think are being referred to?

1 ... protection against fire in hospital or at the doctor's surgery
2 ... protection against theft in hospital or at the doctor's surgery
3 ... protection against misuse of patient data, e.g. unauthorized disclosure of diagnostic data to third parties
4 ... protection against receiving the wrong medical treatment
5 ... protection from complications during medical treatment
6 SPONTANEOUS: other, namely: ....
7 do not know/not specified

Question 2

'Patient safety' is understood to mean the avoidance of unintended or unexpected harm to patients during the provision of health care.

How likely do you think it is that patients in Germany will come to harm as a result of medical treatment in hospital: Do you think it is 'very likely', 'somewhat likely', 'as likely as not', - or 'unlikely'?

1 very likely
2 somewhat likely
3 as likely as not
4 unlikely
5 do not know/not specified

Question 3

And how likely do you think it is that patients will be harmed by medical care provided outside hospital in Germany, e.g. when being treated as an outpatient at a doctor's surgery, or as a result of taking the wrong medication? Do you think it is 'very likely', 'somewhat likely', 'as likely as not', or 'unlikely'?

1 very likely
2 somewhat likely
3 as likely as not
4 unlikely
5 do not know/not specified

Question 4

I will now list possible harmful incidents in medical care, also called 'adverse events'. Please tell me in each case the likelihood you expect such an event happening to you one day by answering: 'yes, definitely', 'yes, probably', 'probably not', or 'definitely not'.
How likely is it that the following will happen to you: ...?

a) ... a dangerous bacterial infection in a hospital
b) ... a wrong diagnosis
c) ... an error during an operation, e.g. a surgical error
d) ... a medication error due to the wrong prescription, dosage or method of application
e) ... an adverse event caused in the use of a medical device

Scale:
1 yes, definitely (incl. has already happened to me)
2 yes, probably
3 probably not

4 definitely not
5 do not know/not specified

Question 5
I will now read the list of 'adverse events' to you again. Please tell me now in each case whether you think that by taking appropriate measures, such events could be largely avoided in the future, by answering: 'yes, definitely', 'yes, probably', 'probably not', or 'definitely not'. Could the following be largely avoided in the future...?

a) ... a dangerous bacterial infection in a hospital
b) ... a wrong diagnosis
c) ... an error during an operation, e.g. a surgical error
d) ... a medication error due to the wrong prescription, dosage or method of application
e) ... an adverse event caused in the use of a medical device

Scale:
1 yes, definitely
2 yes, probably
3 probably not
4 definitely not
5 do not know/not specified

Question 6 (only in 2019)
Who would you contact if you thought a mistake had been made in your healthcare?

1 ... the attending physician or hospital
2 ... another doctor (e.g. general practitioner if he or she was not involved in your treatment)
3 ... a medical association
4 ... your statutory or private health insurance provider, as applicable
5 ... a patient counselling center or patient organization
6 ... a consumer advice center
7 ... a lawyer
8 SPONTANEOUS: Other, namely: ....
9 do not know/not specified

Question 7 (only in 2019)
Health insurance companies have the possibility - based on the data available to them for billing - to recognize whether you, as a patient, have been prescribed medication that could lead to 'adverse events'.

Would you like to be notified by your health insurance company in such a case - or is this not necessary because, for example, the package insert provides sufficient information on side effects?

1 I would like to be notified by my health insurance provider.
2 No notification is necessary.
3 do not know/not specified

Question 8
We have now talked a bit about 'patient safety'. How well informed do you feel you are about patient safety in general: 'very well', 'well', 'acceptably well', or 'not at all'?

1 very well
2 well
3 acceptably well

4 not at all
5 do not know/not specified

Question 9
All in all, do you think that as a patient, you can contribute towards improving the care you receive at the doctor's surgery or in hospital: 'definitely', 'probably', 'perhaps', or 'not at all'?

1 definitely
2 probably
3 perhaps
4 not at all
5 do not know/not specified

Section B

Question 10 (only in 2023)
I am now going to read you some statements around the topic of 'patient safety'. Please tell me in each case whether you ‘completely agree’, ‘rather agree’, ‘rather disagree’ or ’completely disagree” with the statement.

a) I myself take care that no mistakes happen during my treatment in the hospital or in the surgery.
b) It is easy for me to get clear and reliable information about patients' rights and patient safety.
c) I know simple ways of how and where to complain if I have a problem at the doctor's surgery or hospital.
d) Hospitals and practices make it easy for patients to get involved so that mistakes do not happen.

1 agree completely
2 rather agree
3 rather disagree
4 completely disagree
5 do not know/not specified

Question 11 (only in 2023)
Please tell me if you have heard of the following participation opportunities to improve patient safety, or if you have not yet heard of them.

a) feedback forms sometimes given to patients after treatment to find out what they liked or where they see room for improvement
b) quality circles, i.e. regular meetings in which medical staff discuss problems, causes and solutions
c) patient advisory boards or patient committees, which are formed by patients to ensure that medical care is optimally geared to patients' needs
d) reporting systems for critical incidents during treatment, also abbreviated CIRS, i.e. systems to report errors anonymously so that lessons can be learned from them

1 yes, already heard of them
2 no, not yet heard of them
3 do not know/not specified

Question 12 (only in 2023)
How has it been so far when you have expressed critical feedback on patient safety to doctors or nurses? Did you feel you were ‘taken completely seriously’, ‘rather seriously’, ‘rather not taken seriously’ or ‘not taken seriously at all’?

1 taken completely seriously
2 rather taken seriously
3 rather not taken seriously
4 not taken seriously at all
5 SPONTANEOUS: have never expressed critical feedback on patient safety
6 do not know/not specified

Question 13
Now let's talk briefly about the topic of climate change. In general, have you very serious concerns, serious concerns, fewer concerns or no concerns about climate change having an impact on your health?
1 very serious concerns
2 serious concerns
3 fewer concerns
4 no concerns
5 do not know/not specified

Question 14
Due to climate change, there are more and more heat waves in summer, i.e. an unusually large number of days with high temperatures. Would you like your family doctor to advise you whether there is a health risk for you personally due to climate change and how you can better protect your health during heat waves? Please answer one of the following: 'yes, definitely', 'yes, rather', 'no, rather not', or 'no, definitely not'.

1 yes, definitely
2 yes, rather
3 no, rather not
4 no, definitely not
5 do not know/not specified

Question 15
Have you ever experienced at least once or on several occasions that the state of your health was so bad because of the hot weather that you thought you needed medical treatment?

1 yes, on several occasions
2 yes, once
3 no
9 do not know/not specified

Question 16
What do you think: Can heat waves have a negative impact on the following areas? Please answer 'yes, definitely', 'yes, probably', 'no, rather not', or 'no, definitely not'.

a) on the performance of staff in the outpatient sector
b) on the performance of hospital staff
c) on chronic diseases
d) on mental health
e) on the effect of medications
f) on wound healing

Scale:
1 yes, definitely
2 yes, probably
3 no, probably not
4 no, definitely not
5 do not know/not specified

Question 17
What do you think: How well are hospitals in Germany prepared to ensure safe care even during a heatwave? Please answer ‘very well’, ‘well’, ‘not so well’, or ‘poorly’?

Scale:
6 very well
7 well
8 not so well
9 poorly
10 do not know/not specified

Section C

Lastly, we have a few statistical questions that we need to ask you to evaluate the results.

Question S1/age

Question S2/gender

To all
Question S3a
If you now think about the overall state of your health: Would you describe your health in general, as 'very good', 'good', 'satisfactory', 'poor', or as 'very poor'?

1 very good
2 good
3 satisfactory
4 poor
5 very poor
6 do not know/not specified

Question S3b
Are you receiving regular medical treatment for a serious chronic illness?

1 yes
2 no
3 do not know/not specified

Question S3c
Do you regularly take a prescription medication?
INT.: IF 'yes' QUESTION: And how many different medications do you take?

1 one prescription drug
2 two prescription drugs
3 three or more prescription drugs
4 do not know/not specified

Question S4
Are you currently gainfully employed?
Which description best fits your state of employment as defined here...

1 fully employed
2 employed part-time
3 semi-retired
4 in marginal employment (in a ‘mini-job’)

5 working in connection with receipt of a jobseekers' allowance (in a 'one-euro job' as a condition for receiving ALG II)
6 employed occasionally or irregularly
7 in vocational training/an apprenticeship
8 in retraining
9 in military service/civilian service
10 economically inactive

<u>Filter: Only if the answer to item 2, 4, 6, 8, or 10 of question S4 was positive</u>
Question S5
Please tell me which of the following groups you belong to.

1 pupil at school
2 student
3 pensioner, retiree, early retirement
4 unemployed
5 housewife, househusband
6 on maternity leave, parental leave, or other leave of absence
7 not employed as defined, for other reasons
8 none of the above
9 do not know/not specified

<u>To all</u>
Question S6
Educational attainment: What is the highest educational certificate, diploma or degree that you have received?

1 no secondary/vocational school-leaving certificate
2 secondary/elementary school-leaving certificate
3 secondary school-leaving certificate (Realschulabschluss, Fachschulreife)
4 certificate of completion of polytechnic secondary school
5 certificate of advanced technical college entrance qualification; of completion of a specialized upper secondary school
6 certificate of completion of 'A' levels, or subject-linked higher education entrance qualification (Abitur)
7 university degree
8 certificate of another school-leaving qualification

Question S7
What is your main occupation?

1 self-employed farmer or cooperative farmer
2 freelancer
3 self-employed (in a trade, craft, industry, service)
4 civil servant, judge, professional soldier
5 employee
6 worker
7 in training
8 caring for family member(s)
9 other

<u>To all</u>
Question S8a
How many people live permanently in your household, including yourself? Please also consider children living in your household.

/___/ persons

Filter: Only to multi-person households
Question S8b/Children in the household
How many children under the age of 18 live in your household?

/___/ Children

To all
Question S9
What is your marital status? Which of the following applies to you? ...

1 ... married and living with your spouse
2 ... married and living separately from your spouse
3 ... single
4 ... divorced
5 ... widowed
6 no answer

Filter: Only if response to S9 is not 1 'married and living with spouse'.
Question S10
Do you live with a partner in your household?

1 yes
2 no
3 no answer

To all
Question S11
What is the total monthly net income of your household? This is the sum of wages, salaries, income from self-employment, pensions or retirement pensions, in each case after the deduction of taxes and social security contributions. Please also add income from public benefits, income from renting, leasing, housing benefit, child benefit and other income.

1 less than €500
2 €500 up to €1,000
3 €1,000 up to €1,500
4 €1,500 up to €2,000
5 €2,000 up to €2,500
6 €2,500 up to €3,000
7 €3,000 up to €3,500
8 €3,500 up to €4,000
9 €4,000 up to €4,500
10 €4,500 or more
11 not specified

Question S12
Take over federal state/region/location size from the sample.

Thank you very much for taking the time to participate in this interview!
