# Supplementary material for: Patient safety and climate change: findings from a cross-sectional survey in Germany
Source: BMC Public Health. 2024 Nov 21;24:3233. doi: 10.1186/s12889-024-20752-x (PMC11580531; doi:10.1186/s12889-024-20752-x)
Supplement: Supplementary file 2 — Supplementary Material 2. [file 12889_2024_20752_MOESM2_ESM.pdf]

STROBE Statement—checklist of items that should be included in reports of observational studies

	Item No.	Recommendation	Page No.	Relevant text from manuscript
**Title and abstract**	1	(*a*) Indicate the study's design with a commonly used term in the title or the abstract	1	Findings from a cross-sectional survey in Germany
		(*b*) Provide in the abstract an informative and balanced summary of what was done and what was found	1	Abstract
**Introduction**				
Background/rationale	2	Explain the scientific background and rationale for the investigation being reported	2-3	Background
Objectives	3	State specific objectives, including any prespecified hypotheses	4	This study, therefore, aimed to (i) provide data on the public perception of patient safety risks associated with climate change, and (ii) examined the demographic and socioeconomic factors influencing this perception.
**Methods**				
Study design	4	Present key elements of study design early in the paper	4	The TK Monitor of Patient Safety is a large nationally representative survey. It is undertaken yearly and collects population-related data on perceptions, experience, and knowledge pertaining to patient safety from 1,000 randomly selected inhabitants via computer assisted telephone interviews (CATI).

Setting	5	Describe the setting, locations, and relevant dates, including periods of recruitment, exposure, follow-up, and data collection	4	The first survey took place from October to November 2019. The follow-up surveys were conducted in August 2020, in June 2021, in April to May 2022, and in June 2023. Selected results from the surveys have been published elsewhere [17–19]. The data reported here are from 2023.
Participants	6	(*a*) *Cohort study*—Give the eligibility criteria, and the sources and methods of selection of participants. Describe methods of follow-up<br>*Case-control study*—Give the eligibility criteria, and the sources and methods of case ascertainment and control selection. Give the rationale for the choice of cases and controls<br>*Cross-sectional study*—Give the eligibility criteria, and the sources and methods of selection of participants	4	Inclusion criterion was 18 or more years of age (70.37 million adults living in Germany in 2023) and the exclusion criterion was non-German speakers (5% housholds with not sufficient German language skills). We employed a multi-level stratified random sampling procedure. For respondent selection within a household we used the last-birthday method.
		(*b*) *Cohort study*—For matched studies, give matching criteria and number of exposed and unexposed<br>*Case-control study*—For matched studies, give matching criteria and the number of controls per case		n.a.
Variables	7	Clearly define all outcomes, exposures, predictors, potential confounders, and effect modifiers. Give diagnostic criteria, if applicable	5	In section (A) questions on perceptions, experiences, and subjective information relating to patient safety in medical care

				were scored on a Likert scale, from very likely to unlikely. The questions in section (B) covered perceptions and knowledge regarding climate change. In section (C) sociodemographic and socioeconomic data were collected. Section A and C remained almost unchanged every year, while section B changed from survey to survey. The data reported here are from sections B and C.
Data sources/ measurement	8*	For each variable of interest, give sources of data and details of methods of assessment (measurement). Describe comparability of assessment methods if there is more than one group	4	Self-reported data from nationally representative samples of 1,000 adults were collected.
Bias	9	Describe any efforts to address potential sources of bias	4	The contact data and quotas including response rate were deleted immediately after the interviews.
Study size	10	Explain how the study size was arrived at	4	The TK Monitor of Patient Safety is a large nationally representative survey. It is undertaken yearly and collects population-related data on perceptions, experience, and knowledge pertaining to patient safety from 1,000 randomly selected inhabitants

Quantitative variables	11	Explain how quantitative variables were handled in the analyses. If applicable, describe which groupings were chosen and why	5	Data were weighted for gender, age, education level, and area (metropolitan/rural) population distribution. Weighting was based on iterative proportional fitting.
Statistical methods	12	(*a*) Describe all statistical methods, including those used to control for confounding	5	Ordinal logistic regression was used to determine the factors influencing respondents' perception about climate change, and odds ratios (OR) were calculated.
		(*b*) Describe any methods used to examine subgroups and interactions		n.a.
		(*c*) Explain how missing data were addressed	5	We originally intended to include self-reported professional positions, and marital status/household structure as covariates, but 50% of survey respondents did not answer these questions.
		(*d*) *Cohort study*—If applicable, explain how loss to follow-up was addressed<br>*Case-control study*—If applicable, explain how matching of cases and controls was addressed<br>*Cross-sectional study*—If applicable, describe analytical methods taking account of sampling strategy		n.a.
		(*e*) Describe any sensitivity analyses		
**Results**				
Participants	13*	(a) Report numbers of individuals at each stage of study—eg numbers potentially eligible, examined for eligibility, confirmed eligible, included in the study, completing follow-up, and analysed	4	Inclusion criterion was 18 or more years of age (70.37 million adults living in Germany in 2023) and the exclusion criterion was non-German speakers (5% housholds with not sufficient German language skills).

		(b) Give reasons for non-participation at each stage		
		(c) Consider use of a flow diagram		
Descriptive data	14*	(a) Give characteristics of study participants (eg demographic, clinical, social) and information on exposures and potential confounders		Table 1
		(b) Indicate number of participants with missing data for each variable of interest	4	The contact data and quotas including response rate were deleted immediately after the interviews.
		(c) *Cohort study*—Summarise follow-up time (eg, average and total amount)		
Outcome data	15*	*Cohort study*—Report numbers of outcome events or summary measures over time		Table 1
		Case-control study—Report numbers in each exposure category, or summary measures of exposure		
		Cross-sectional study—Report numbers of outcome events or summary measures		
Main results	16	(*a*) Give unadjusted estimates and, if applicable, confounder-adjusted estimates and their precision (eg, 95% confidence interval). Make clear which confounders were adjusted for and why they were included		Table 2-4
		(*b*) Report category boundaries when continuous variables were categorized		
		(*c*) If relevant, consider translating estimates of relative risk into absolute risk for a meaningful time period		
Other analyses	17	Report other analyses done—eg analyses of subgroups and interactions, and sensitivity analyses		
**Discussion**				
Key results	18	Summarise key results with reference to study objectives	11	This study showed a high perception of the health threat of climate change among the general German population. Our study is the first study assessing perceptions, experiences, and knowledge relating to patient safety and climate change in Germany.
Limitations	19	Discuss limitations of the study, taking into account sources of potential bias or imprecision. Discuss both direction and magnitude of any potential bias	12	Strengths and limitations of the study

Interpretation	20	Give a cautious overall interpretation of results considering objectives, limitations, multiplicity of analyses, results from similar studies, and other relevant evidence	12	Strengths and limitations of the study
Generalisability	21	Discuss the generalisability (external validity) of the study results	12	Furthermore, as the contact database was deleted immediately after the information had been collected, in accordance with data protection regulations, the response rate and reasons for non-participation are unknown. This has implications for the generalizability of the findings. The views of people who do and do not participate may herein differ. However, the sample was nationally representative for German adolescents, and the sample size was large and statistically robust.
**Other information**				
Funding	22	Give the source of funding and the role of the funders for the present study and, if applicable, for the original study on which the present article is based	13	Funding: The TK Monitor of Patient Safety was financed by the statutory health fund 'Techniker Krankenkasse'

*Give information separately for cases and controls in case-control studies and, if applicable, for exposed and unexposed groups in cohort and cross-sectional studies.

Note: An Explanation and Elaboration article discusses each checklist item and gives methodological background and published examples of transparent reporting. The STROBE checklist is best used in conjunction with this article (freely available on the Web sites of PLoS Medicine at http://www.plosmedicine.org/, Annals of Internal Medicine at http://www.annals.org/, and Epidemiology at http://www.epidem.com/). Information on the STROBE Initiative is available at www.strobe-statement.org.
